# Organic Synthesis and Catalysis Enable Facile Access to Bioactive Compounds and Drugs

**DOI:** 10.1021/acscentsci.4c02041

**Published:** 2024-12-16

**Authors:** Svetlana B. Tsogoeva, Kirk S. Schanze

The process of drug discovery and development is inherently complex, resource-intensive, and multidisciplinary. *Organic synthesis* and *catalysis* play key roles in transforming this process by enabling the efficient construction of bioactive compounds and pharmaceuticals.

*Total organic synthesis* remains a fundamental aspect of organic chemistry, allowing the generation of complex natural compounds and bioactive molecules while driving drug discovery and development. Recent advancements in the field have demonstrated innovative new strategies for synthesizing novel therapeutics, e.g., anti-inflammatory compounds, treatments for osteoporosis, and antiviral agents with enhanced efficacy.^1−6^

Cutting-edge approaches to organic synthesis include *enzyme-, transition metal-, photo-,* and *organocatalysis*, which are instrumental in accelerating the discovery of new drug candidates. Recent advances in *enzyme catalysis* have enabled substrate-selective catalysis and chemoenzymatic methods, facilitating the efficient synthesis of natural products and pharmaceuticals with enhanced regio- and stereoselectivity. These recent developments demonstrate the growing importance of enzyme-catalyzed transformations in medicinal chemistry, providing green, scalable routes to therapeutic compounds.^7−12^ Catalytic techniques, such as *transition metal-, photo-,* and *organocatalysis*, have significantly broadened further the scope of bond forming reactions. Key recent developments include stereoselective metal-catalyzed additions and C–H functionalizations, organo- and photocatalyzed transformations, and boron-centered radical reactions, all of which have advanced synthetic applications. These functional group transfer strategies are enabling late-stage diversification, and creating valuable bioactive compounds, demonstrating the high impact of catalysis on drug discovery and medicinal chemistry.^13−20^

**Figure 1:**
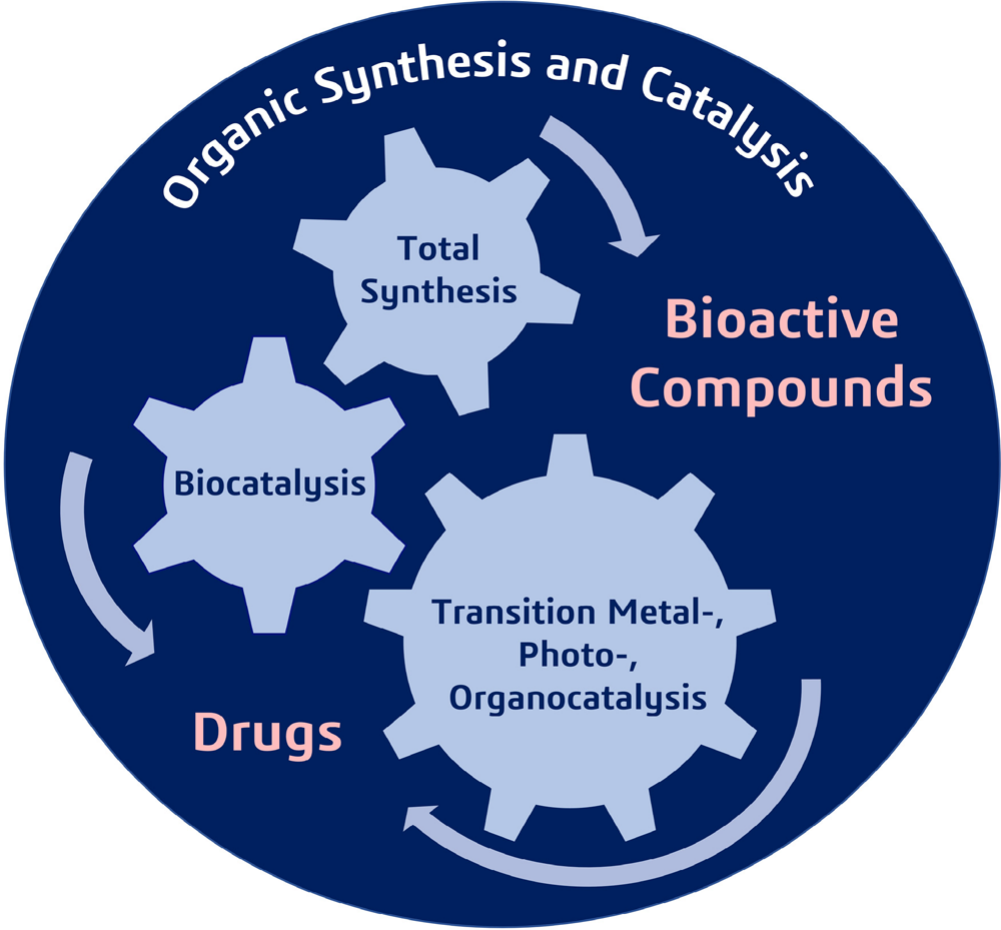
Collective synthetic efforts toward facile access to bioactive compounds and drugs.

This Collection highlights the latest advances in organic synthesis and catalysis that have been published recently in *ACS Central Science*, demonstrating strategies for efficient and selective construction of pharmaceutically relevant compounds, natural products, and drugs. Emphasizing impactful work in total synthesis, biocatalysis, and catalytic methodologies—including transition metal-, photo-, and organocatalyzed reactions—this Collection reflects the cutting-edge research in the field of organic chemistry related to medicinal chemistry.

## Total Synthesis

The total synthesis of bioactive compounds remains a cornerstone of organic chemistry, providing not only the means to access complex natural products but also the opportunity to explore new chemical scaffolds with potential therapeutic applications. Recent advances in the synthesis of bioactive molecules have demonstrated the key role that such efforts play in the discovery and optimization of compounds targeting a variety of diseases.

In this context, **Wang, Gao**, and co-workers recently reported two novel phthalides, falcarinphthalides A and B, from *Angelica sinensis*, with falcarinphthalide A showing potent antiosteoporotic activity by inhibiting NF-κB and c-Fos signaling.^1^ They successfully achieved a bioinspired gram-scale total synthesis of falcarinphthalide A, offering a promising new scaffold for osteoporosis treatment. Recent advancements in the structural modification of cannabinoid receptor type 2 (CB_2_R) ligands and selective inverse agonists have significantly enhanced our understanding of their therapeutic potential in managing inflammatory conditions. **Frank, Grether, Carreira**, and their teams presented the structure-based design of selective cannabinoid receptor type 2 (CB_2_R) inverse agonists, derived from the agonist HU-308 by modifying the side chain to introduce a phenyl group.^2^ The lead compound exhibits high affinity for CB_2_R and serves as a versatile platform for creating fluorescent probes that retain inverse agonist activity, stabilizing CB_2_R in its inactive state without activating key signaling pathways. Furthermore, innovations in synthetic methodology, such as the development of a concise route to salvinorin analogs targeting the kappa-opioid receptor (KOR), were reported. **Bohn, Shenvi**, and co-workers presented an elegant short asymmetric synthesis of salvinorin analogs, leveraging a sterically confined organocatalyst and cobalt-catalyzed cycloaddition to access a focused library of compounds.^3^ The resulting analogs demonstrate enhanced potency, selectivity, and functional bias at the kappa-opioid receptor (KOR), surpassing the properties of salvinorin A, offering the potential for next-generation analgesics and other therapeutic applications. In the antiviral field, the efficient synthesis of antiviral candidates and the scalability and environmental considerations are crucial for large-scale drug generation. Along this line, the group of **Kawajiri** outlined the development of a scalable, efficient manufacturing process for the SARS-CoV-2 antiviral candidate Ensitrelvir, focusing on the convergent synthesis of key indazole, 1,2,4-triazole, and 1,3,5-triazinone fragments.^4^ The optimized process improved the yield 7-fold, enhanced intermediate stability with a meta-cresolyl moiety, and minimized the environmental impact by using direct crystallization for intermediate isolation, reducing solvent and reagent waste. Additionally, biomimetic approaches, such as the macrocyclization strategies, were also successfully employed in the synthesis of natural products. **Hong** and co-workers reported the first biomimetic total synthesis of chejuenolides A–C, based on a hypothetical Mannich macrocyclization, using a lactone-based precursor constructed via aldol–Julia–aldol reactions.^5^ The synthesis revealed stereochemical insights, showing that the β-oxo-δ-lactone unit easily converts to C2/C18 diastereoisomers, providing key information about stereoselectivity in the proposed enzymatic biosynthetic pathway. Finally, **Li, Patil**, and their teams reported the total synthesis and structure–activity relationship exploration of laterocidine, a cyclic lipodepsipeptide with potent activity against multidrug-resistant Gram-negative pathogens.^6^ The work identified key structural features responsible for its antimicrobial action and led to the development of an engineered peptide with enhanced efficacy, including complete inhibition of polymyxin-resistant *Pseudomonas aeruginosa*.

Together, these remarkable examples illustrate the continued power of total synthesis in advancing medicinal chemistry and drug development.

## Biocatalyzed Reactions

Biocatalysis has emerged as a highly useful approach in the synthesis of a diverse variety of compounds of interest for medicinal chemistry, leveraging recent advances in enzyme technology. Enzyme catalysis can serve as a crucial step in the total synthesis of bioactive compounds, facilitating highly selective transformations that enhance efficiency and yield while minimizing the use of harsh reagents.

Along these lines, the **Narayan** group employed substrate-selective catalysis to direct the final cyclization of intermediates, allowing the synthesis of azaphilone natural products with linear or angular tricyclic cores.^7^ By utilizing a flavin-dependent monooxygenase (FDMO) and acyl transferase (AT) in sequence, the method enabled the efficient total synthesis of five azaphilone natural products and several unnatural derivatives in a single reaction vessel. Recently, the group of **Li** presented the total synthesis of the tumor-associated glycolipid disialosyl globopentaosylceramide (DSGb5) using a chemoenzymatic approach.^8^ Through regio- and stereoselective enzyme-catalyzed sialylation, the challenging α2,6-linked sialoside was installed, and binding studies revealed that DSGb5 exhibits higher affinity for Siglec-7 than its oligosaccharide moiety, highlighting the role of the ceramide in enhancing multivalent interactions for recognition. Additionally, advances such as enzyme encapsulation in metal frameworks and directed evolution of enzyme variants further demonstrate the potential of biocatalysis in constructing intricate chiral molecules and advancing cancer therapeutics. **Yuan, Zhang, Cheng**, and their teams reported a green synthesis strategy for encapsulating enzymes within metal azolate frameworks (MAFs) using micelles, significantly enhancing the catalytic efficiency of enzymes like BCL for the asymmetric synthesis of larger chiral molecules.^9^ By optimizing pore sizes and surfactants, the resulting BCL@MAF-6-SDS catalyst showed 420 times higher efficiency than ZIF-8, achieving 94–99% enantioselectivity and near-quantitative yields for drug precursor synthesis. Biocatalytic platforms for constructing chiral N-heterocycles underscore the potential of engineered enzymes in synthetic chemistry. In their recent study, **Arnold** and co-workers presented an enzymatic platform for the biocatalytic construction of chiral N-heterocycles, specifically pyrrolidines and indolines, via intramolecular C(sp^3^)–H amination of organic azides.^10^ By applying directed evolution to cytochrome P411 variants, they developed enzymes capable of selectively inserting alkyl nitrenes into C(sp^3^)–H bonds, demonstrating efficient enantioselective synthesis of these important building blocks and highlighting the potential of new-to-nature biocatalysis in complex molecule construction.

Enzymes have also been leveraged in biocatalytic cascades to produce versatile bioactive scaffolds. Along this line, the group of **Flitsch** reported a protecting-group-free chemoenzymatic and biocatalytic cascade for the efficient synthesis of iminosugars, reducing the process to two steps with over 70% product yield.^11^ By using galactose oxidase and the promiscuous activity of bacterial shikimate dehydrogenases, the approach offers a scalable, one-pot method for producing highly polar iminosugar scaffolds, which are important pharmaceutical targets. Another valuable chemoenzymatic approach to synthesize cepafungin I and its analogues, aiming to better understand the structure–activity relationship of the potent proteasome inhibitors with cancer treatment potential, was reported by **Adibekian, Renata**, and co-workers.^12^ Through the synthesis of 13 analogues and chemoproteomic studies, five were found to be more potent than the natural product, with one analogue exhibiting 7-fold greater inhibition of the proteasome β5 subunit, showing promising activity against multiple myeloma and mantle cell lymphoma compared to the clinical drug bortezomib.

These strategies illustrate the power of enzyme-based methods in the synthesis of biologically active compounds with potential therapeutic applications.

## Transition Metal-, Photo-, and Organocatalyzed Reactions

Catalytic organic synthesis plays a pivotal role in advancing modern chemistry by enabling efficient, selective, and sustainable methods for constructing complex molecular architectures. Recent advances in catalysis, including *transition metal-, photo-,* and *organocatalyzed* reactions, have opened new avenues for bond formation, transforming previously inert functional groups and expanding the scope of organic reactions.

In their recent study, **Zhang** and co-workers presented a novel strategy for the stereoselective 1,4-syn-addition to cyclic 1,3-dienes using hybrid palladium catalysis, offering broad substrate tolerance and mild conditions.^13^ The method enables the efficient synthesis of bioactive molecules, including a TRPV6 inhibitor and CFTR modulator, with a highly selective radical-polar crossover mechanism (dr > 20:1). Recently, the group of **Lu** introduced a novel Cu/Cr catalytic system that enables the direct functionalization of inert alkyl C–H bonds by converting them into nucleophilic alkyl–Cr(III) species at room temperature.^14^ This strategy facilitates carbonyl addition reactions and 1,1-difunctionalization of aldehydes under mild conditions, offering a versatile method for synthesizing aryl alkyl alcohols and other complex molecules. A notable recent development in the field of catalysis is the use of boron-centered radicals. The **Wang** group unveiled a novel approach for generating aryl radicals from tetraarylborate salts via boron-centered radicals using a simple activation reagent.^15^ The method enables the formation of C–B, C–C, and C–X bonds under visible light, broadening the synthetic applications of boron radicals in organic transformations. Similarly, the advent of Pd-catalyzed C–H glycosylation reactions has provided efficient pathways for synthesizing C-glycosides. **Yu, Lei**, and their teams introduced a new Pd-catalyzed C–H glycosylation method that enables the efficient synthesis of C-glycosides by coupling native carboxylic acids with glycals, without external directing groups.^16^ The approach, applied to different substrates, led to the discovery of a potent SGLT-2 inhibitor with antidiabetic potential, manifesting its utility in drug discovery and late-stage diversification. Another breakthrough involves the selective transformation of methyl groups in natural products. **Hartwig** and co-workers presented a novel strategy for selectively transforming methyl groups in terpenoids via C–H bond functionalization, which enabled substitution, elimination, or integration into the molecular skeleton through C–C bond cleavage.^17^ This approach expands the synthetic utility of methyl groups, allowing for the formation of complex architectures and functional derivatives with relevance to medicinal chemistry. A recent work by the group of **Hu** introduced a nickel-catalyzed method for the enantio- and diastereoselective synthesis of fluorinated compounds with vicinal stereogenic centers, without the need for directing groups.^18^ The approach enables efficient access to highly enantioenriched organofluorine compounds and vicinal difluorides. The teams of **Dai** and **Lu** reported a novel skeletal recasting strategy for molecular editing of pyrroles, enabling the transformation of simple pyrroles into fully substituted pyrroles via a phosphoric acid-promoted one-pot reaction.^19^ The method facilitates the construction of tetrasubstituted pyrroles, N–N axial chirality, and the synthesis of the anticancer drug Sutent, with potential applications to other heterocycles.

Finally, **Qi**, **Wang**, and co-workers presented a novel catalytic asymmetric three-component radical cascade reaction using synergistic photoredox and Brønsted acid catalysis, enabling the formation of enantioenriched α-amino acid derivatives with high stereoselectivity.^20^ The reaction, involving radical addition, ring-opening, and radical–radical coupling, offers an efficient method for constructing new valuable molecules under mild conditions, supported by mechanistic studies and quantum calculations.

Overall, the intricate and transformative role of organic synthesis and catalysis in drug discovery and development smoothly aligns with the overarching scope of the *ACS Central Science*, which covers a broad range of topics across the chemical sciences, with a focus on high-impact, multidisciplinary research that connects chemistry to various fields. The set of articles in this Collection provides outstanding examples of the leading research in the field of organic chemistry with applications to biology and medicinal chemistry that have been published in *ACS Central Science* over the past three years. The link between fundamental advances in organic chemistry and catalysis, and progress in bio- and medicinal chemistry applications is evident, making these papers an excellent fit for the scope of *ACS Central Science*. The editors of the journal are enthusiastic to review outstanding papers that represent interdisciplinary research in the chemical sciences and allied fields, and authors working in all areas of the chemical sciences are encouraged to submit their excellent manuscripts to the journal.

In closing, we hope you enjoy reading this special Collection covering total synthesis and innovative catalytic methods, which exemplify the dynamic progress in organic synthesis, offering new tools for building complex structures with precision and efficiency while contributing significantly to drug discovery, medicinal chemistry, and the broader field of organic chemistry.
